# The influence of interoceptive accuracy on the verbalization of emotions

**DOI:** 10.1038/s41598-023-49313-9

**Published:** 2023-12-13

**Authors:** Naho Suzuki, Tetsuya Yamamoto

**Affiliations:** 1 Graduate School of Human Relations, Keio University, Tokyo, 108-8345 Japan; 2 Graduate School of Technology, Industrial and Social Sciences, Tokushima University, Tokushima, 770-8502 Japan

**Keywords:** Psychology, Health care

## Abstract

Interoception, which pertains to the physiological state of the body, is associated with subjective emotional experiences. In particular, the accuracy of perceiving interoceptive signals (interoceptive accuracy [IAcc]) is linked to the intensity of emotional arousal, known as arousal focus (AF). IAcc is believed to influence the granularity of emotional experiences. Here, we examined the relationship between IAcc and assessment and verbalisation of one's own or others' emotions. Study I demonstrated that individuals with higher IAcc exhibited significantly greater AF when evaluating their own positive emotions. Furthermore, although no correlation between IAcc and AF was found in free descriptions of emotions, a significant positive correlation was found between IAcc and the number of emotion-related words. Study II showed that individuals with higher IAcc displayed significantly higher AF when assessing the positive emotions of characters in videos. Additionally, in free descriptions of these characters, a significant positive correlation was observed between predicted verbal IQ and the number of emotion-related words. These findings support the notion that interoception is associated with AF during assessment of one's own or others' positive emotions as well as the abundance of emotion-related words. This study demonstrates the relationship between bodily sensations and social aspects of human embodiment.

## Introduction

Sensation pertaining to the internal physiological state of one's body is referred to as interoception^1,2^. Interoception is a term first coined by Sherrington^1^. It is currently defined as the sensation of changes in the body’s internal state, composed of signals transmitted from organs, skin, muscles, and other bodily sources to the brain. Neuroimaging studies have demonstrated activation in various brain regions, including the insular cortex, anterior cingulate cortex, and thalamous, when individuals consciously perceive interoception^3,4^. Notably, these brain regions are also involved in the neural underpinnings of subjective emotional experiences^5^. From these studies, it has been hypothesized that integration of interoception with the current contextual and environmental information serves as the foundation for subjective emotional experiences, and the prcess of becoming aware of one’s emotions is assumed to include referencing one’s own interoception^6^. Given that interoception is considered one of the elements contributing to the generation of emotions, the ability to accurately perceive interoception would influence the intensity of experienced emotions. In fact, it has been suggested that interoceptive accuracy (IAcc) is related to mood states^5^.

In emotion research, emotions are commonly conceptualised along two dimensions: valence and arousal. The magnitude of the influence of arousal on emotional recognition is referred to as arousal focus (AF), whereas the magnitude of the influence of valence is known as valence focus (VF). Concepts similar to AF include emotional granularity, which describes an individual's ability to create experiences of emotion that are precise and context-specific^7^, is defined by the degree of AF and VF^8,9^. Among these concepts, interoception has been associated with arousal, as individuals with high IAcc tend to evaluate arousal ratings for both positive and negative stimuli as significantly higher than do individuals with low IAcc^10^. These findings suggest that interoception is involved in the generation of emotions and that IAcc, which reflects the extent to which interoceptive sensations are perceived, is related to emotional arousal. Furthermore, core elements of social cognition, such as theory of mind^11^, empathy^12^, and imitation^13^, have all been shown to be related to interoception, indicating that individuals with high IAcc possess a greater capacity to understand others' mental states.

While interoception plays a role in directing attention towards and understanding one's own and others' emotions, recent research has extended the investigation of the relationship between interoception and emotions to include connections with language when expressing emotions. For example, Lisa et al.^8^ has examined this relationship using an emotion rating task. Previously, basic emotions such as anger, happiness, and fear were considered universally innate across humans. However, a new hypothesis challenges the universality of emotions and suggests that emotions are constructed through language^14^. Consequently, the association between language and emotions has gained attention from a neuroscientific perspective, and higher IAcc has been found to be associated with greater AF^8^. These insights suggest connections among interoception, emotions, and language, highlighting the interplay between bodily experiences and the linguistic expression of emotions within social interactions^15^.

Nevertheless, the relationship between interoception and the characteristics of the language used to infer and express others' emotions in interactive settings has not been clearly elucidated. Furthermore, the relationship between features of spontaneous speech and interoception has not yet been examined. In daily life, individuals imagine others' emotions in order to control their own behaviour. Therefore, by investigating how one’s interoception relates to the recognition and verbalisation of others' emotions, we can illuminate the impact of bodily experiences on higher-order communication functions. Therefore, the aim of this study is to clarify the influence of one's own level of IAcc on the characteristics of the language used to express one’s own and others' positive and negative emotions. Using first-person-perspective videos allows for participants to rate their ow emotions while viewing the video and describe their own emotions freely. This approach enables an examination of the relationship between IAcc and the linguistic characteristics when expressing their own emotions. Similary, the use of thir-person-perspective videos allows participants to estimate and rate the emotions of the character within the video and describe characters’ emotions freely. This methodology facilitates the investigation of the relationship between IAcc and the linguistic features used when inferring the emotions of others. Therefore, in this study, both first-person-perspective and third-person-perspective videos were utilized.

We examine this issue through two studies and hypothesize that when rating and freely describing their own and others’ positive and negative emotions, the individual with higher IAcc shows higher AF. We also hypothesize that the individual with higher IAcc use more emotional words when freely describing their own and others’ emotions. Besides, we exploratory examine that how the level of IAcc influences the characteristics of word-to-word connections and the differences in descriptive content when freely describing one’s own and others’ emotions by text mining. Previous reseraches have suggested that greater IAcc is related to greater AF, hence we hypothesized that individuals with high IAcc shows diverse word-to-word connections.

## Methods

### Participants and data collection

In this study, data from thirty-nine undergraduate students and graduate students (15 males, 24 females; mean age = 21 years, *SD* = 1.48) were analysed. They were all Japanese students and native Japanese speakers. In this study, we recruited participants from university students to examine the impact of IAcc on linguistic features, as it is less likely to be influenced by significant differences in language proficiency or memory difficulties due to aging. Besides, previous studies have shown that those with high alexithymia tendencies have unique characteristics of interoception^2,3,16^. The aim of this study is to examine the relationship between IAcc and language in a group of healthy students. Given the potential for alexithymia to confound these relationships, it has been excluded from this study. In addition, given the possibility that personality may influence the dialogue^17^, we used the Japanese version of the Ten Item Personality Inventory (TIPI-J)^18^ to control for this influence.

We recruited participants after the class ended. Then, we explained that there would be no disadvantages, such as affecting their grades in the class, for those who chose not to participate in the experiment. Experiments were conducted between October 2022 and January 2023. We used G*Power^19,20^ to determine the sample size. When setting a two-tailed test with an effect size of 0.5, an α error of 0.05, and a 1-β error of 0.8, it was determined that the required sample size was 26. Consequently, it can be inferred that the sample size for this study is deemed sufficient. This study was approved by the Research Ethics Committee of the Graduate School of Social and Industrial Sciences and Technology, Tokushima University (acceptance number: 267). It was performed according to the ethical standards of the 1964 Declaration of Helsinki and its amendments.

### Materials

#### Videos

We selected and used 60 videos with a Creative Commons licence uploaded to the video-sharing platforms YouTube and FilmStim^21^. FilmStim, created by Pierre Mahau, is a collection of film clips that effectively elicit various types of emotions^22^. All videos had a duration of approximately 30 s. Study I focused on data obtained from first-person-perspective videos, whereas Study II focused on data obtained from third-person-perspective videos. Ten positive, neutral, and negative videos were selected from each category. The videos were accompanied by audio containing human voices and ambient sounds. The human voices were in Japanese.

In order to select videos that do not exhibit a bias in emotional valence variance for both first-person-perspective and third-person-perspective videos, we conducted a preliminary experiment. Five Japanese students (all female, Mean Age = 23.4, *SD* = 1.02) participated in the experiment. After viewing the videos, they rated the extent to which the emotions evoked by each video were positive on a 10-point Likert scale ranging from 1 (not at all) to 10 (extremely). Similary, they also rated the extent to which the emotions evoked by each video were negative on a a 10-point Likert scale. We used a total of 60 videos, consisting of 30 first-person-perspective videos and 30 third-person-perspecitve videos. For each set of videos, we preselected 10 videos each that were expected to evoke positive emotions, neutral emotions, and negative emotions. To compare the positive and negative scores, we conducted Friedman test and further performed Wilcoxon sined-rank tests with Bonferroni correction for multiple comparisons.

The results of the analysis revealed significant differences in the positive scores for first-person-perspective videos between positive and neutral videos, positive and negative videos, and neutral and negative videos (*p*s < 0.03). The positive scores were found to be significantly higher in the order of positive, neutral, and negative videos. Regarding the negative scores, significant differences were observed between negative and positive videos, and negative and neutral videos, with negative videos having higher negative scores than positive and neutral videos (*p*s < 0.02). For third-person-perspective videos, there were significant differences in the positive scores between positive and neutral videos, and positive and negative videos, so positive videos had significantly higher positive scores compared to neutral and negative videos (*p*s < 0.02). In terms of negative scores, significant differences were observed between negative and positive videos, negative and neutral videos, and positive and neutral videos (*p*s < 0.02). Negative scores were significantly hifher in the order of negative, neutral, and positive videos. In conclusion, all categories were supposed to be appropriately classified based on emotional valence.

#### Language ability

The Japanese Adult Reading Test (JART)^23^ was used to control for the influence of language abilities. The JART is a standardised test developed by Matsuoka et al.^23^ based on the findings of the National Adult Reading Test (NART)^24^ and adapted for the Japanese population^25^.

#### lnteroceptive accuracy

IAcc was assessed via the commonly used mental tracking method^26^, namely the heartbeat counting task. The smartphone application “HRV: Heart Rate Cardio Monitor” (https://apps.apple.com/us/app/hrv-heart-rate-cardio-monitor/id1456358655) was used for this task. Participants place their index finger on the camera portion of the smartphone to be used. The experimenter verbally signals the start and end of the measurement, during which the participants count their own pulse rate. Parricipants do not actually measure their pulse rate by placing their fingers on their wrists, but counted it by their own senses. After the end of the trial is signalld, pariticipants verbally tell the experimenter the number of pulses they have counted. This was repeated three times. IAcc was calculated using the following formula^27^:

$$ {\text{IAcc }} = {{ 1}/{3 }}\,{^*} \, \sum {\left( {{1} - |{\text{actual heartbeat count}} - {\text{reported heartbeat count}}|} \right)/{\text{actual heartbeat count}}} {.} $$

### Scales

#### Alexithymia

The Japanese version of the 20-item Toronto Alexithymia Scale (TAS-20)^28^ was utilised in this study. The TAS-20 measures three sub-dimensions: difficulty identifying feelings (difficulty in recognising and distinguishing emotions and associated bodily sensations), difficulty describing feelings (difficulty in verbalising emotions to others), and externally oriented thinking (tendency to focus on external facts). It has been widely used interinationaly with high reliability and validity^28^. The scale consists of 20 items and participants were asked to respond to each item using a scale ranging from 1 (not at all applicable) to 5 (very applicable). The scale has a cutoff point, with a score of 61 or higher indicating alexithymia.

#### Personality

The Japanese version of the Ten-Item Personality Inventory (TIPI-J)^18^, created by Gosling et al. (2003)^29^, was employed in this study. The scale consists of 10 questions corresponding to each factor of the Big Five personality traits. It has been widely used internationaly with high reliability and validity^18^. Participants were asked to rate their agreement with each of the items on a 7-point Likert scale ranging from 1 (strongly disagree) to 7 (strongly agree).

### Procedure

**Figure 1:**
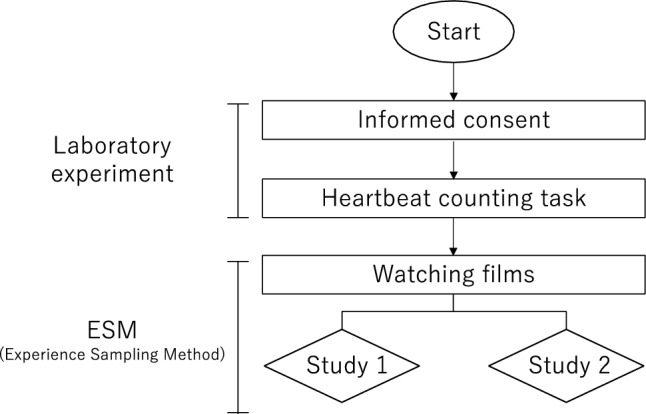
Flow of an experiment.

The overall flow of Study I and Study II is as follows (see Fig. 1). At first, screening surveys were conducted to exclude participants who met any of the exclusion criteria: presence of arrhythmia, history of heart disease or mental disorder, regular yoga or mindfulness training, non-native Japanese speakers, and TAS-20 score ≥ 61. As a preliminary survey, the participants were asked to respond to the TIPI-J to measure their personality traits.

The participants were invited to the laboratory, where informed consent was obtained. Subsequently, JART and heartbeat counting tasks were conducted and the order of these tasks were counterbalanced. Following an explanation of the upcoming video viewing and questionnaire responses, and upon obtaining informed consent, the laboratory experiment was concluded. To ensure ecological validity, the data collection methods for Studies I and II adopted the experience sampling method (ESM) rather than traditional laboratory experiments. ESM involves collecting measurements from participants in their daily lives over several days, multiple times a day, at scheduled or random time points^30^. ESM offers several advantages over other methods that collect data in a single or a limited number of sessions^31^, including its high ecological validity, minimal recall bias, and high temporal resolution. The participants were instructed to watch two videos and provide free-text descriptions and ratings for each video using Google Forms, which were sent via email to the researchers. This process was conducted for 30 days. The videos and response forms were randomly sent between 10 AM and 8 PM, with one fixed time slot during which the participants could concentrate on the task. Participants were instructed to watch the randomly sent videos and provide responses within two hours of receiving the notification whenever possible. We analysed Study 1 data using first-person-perspective videos and Study 2 data using third-person-perspective videos.

#### Study I

After watching the videos, the participants were first asked to provide a free-text description of their emotions following the video. In order to analyze the participants’ spontaneous free-responses, no time limit was set for their responses, and the instructional text stated, “Please answer freely as you see fit.”. In addition, they were asked to rate the extent to which nine emotional words (happiness, joy, engagement, fun, tension, anger, sadness, embarrassment, and guilt) described their emotions after watching the video. This rating was executed using a 7-point scale ranging from ‘not at all’ (score = 0) to ‘very much’(score = 6)^32^.

#### Study II

After watching the videos, the participants were asked to provide a free-text description of the emotions they believed the characters in the videos were experiencing. For the same reasons as in Study I, the instructional text stated, “Please answer freely as you see fit.”. Besides, they were asked to rate the extent to which the nine emotional words (happiness, joy, engagement, fun, tension, anger, sadness, embarrassment, and guilt) described the emotions of the characters in the videos. These ratings were executed using the same response scale as in Study I^32^.

### Analyses

For all tests, significance was set at α= 0.05 (two-tailed). SPSS version 27.0, RStudio version 3.6.0^33^, Java version17.0.5^34^, Python2.7^35^, and KH Coder version3. Alpha17e^36,37^ were used for the analyses. After calculating the descriptive statistics for each scale score of participants, the following analyses were conducted.

#### AF from rating data

To calculate the degree of AF toward positive emotions (positive AF), the rating data of four positive emotion adjectives (happiness, joy, engagement, and fun) were used. Similarly, to calculate the degree of AF toward negative emotions (negative AF), the rating data for five negative emotion adjectives (tension, anger, sadness, embarrassment, and guilt) were used. Emotional granularity, a concept relevant to this study, is typically operationally defined as the extent to which arousal ratings for various emotional descriptive adjectives co-vary across ratings^38,39^. Previous studies have used the intraclass correlation coefficient (ICC) as an index of AF^40^. ICC can be used to reflect the agreement among emotional states across measurement periods and capturing temporal consistency^38^. However, Shapiro-Wilk tests revealed a lack of normality of the rating data for positive and negative AF. Therefore, Fleiss' kappa coefficient, a measure of agreement among three or more raters, was used as an index of AF in this study. A larger Fleiss' kappa coefficient indicates that different emotion adjectives are used in the same manner to describe emotional experiences, suggesting less emotional granularity and lower AF. Conversely, a smaller Fleiss' kappa coefficient indicates that different emotion adjectives are used in different ways to describe emotional experiences, suggesting greater emotional granularity and higher AF. Thus, in this study, Fleiss' kappa coefficients for the four positive emotion adjectives (happiness, joy, engagement, and fun) were calculated for each participant to determine positive AF. Similarly, Fleiss' kappa coefficients for the five negative emotion adjectives (tension, anger, sadness, embarrassment, and guilt) were calculated for each participant to determine negative AF. Correlations were calculated between the ratings of positive AF, negative AF, and IAcc.

#### AF from free description data

To calculate AF from the free-text responses, the Japanese version of SentiStrength, namely ‘japanese-sentistrength’ (https://github.com/tiffanywhsu/japanese-sentistrength), was used^41^. SentiStrength is an emotion analysis package that labels the intensity of positive and negative emotions in text. It was developed specifically to analyse emotions in short texts on social media. The Japanese version of SentiStrength was created based on the English version and allows for the scoring of short Japanese texts based on the dimensions of emotional valence (positive/negative) and arousal (1–5). The degree of positive sentiment in a sentence is expressed on a scale of 1–5, while the degree of negative sentiment is expressed on a scale of − 1 to − 5. In this study, positive and negative scores were calculated for each sentence. The reliability and validity have been shown^41^. The standard deviation (SD) of the positive and negative scores of all the data was then computed as a measure of the variability in positive and negative sentiment within the free-text data. These values were used as indicators of positive and negative AF, respectively. Correlations were calculated between positive AF, negative AF, and IAcc, using the free-description data.

#### Text mining using free description data

The text mining analysis was conducted using KH Coder^36,37^. First, the relationship between the total number of extracted words and the number of words related to emotions within a single sentence was examined in relation to IAcc. To determine the number of words related to emotions, a list of the 150 most frequent words was obtained. The number of words related to emotions in the list was calculated. The Emotional Expression Dictionary^42^ was consulted to select emotion-related words. In Japanese emotion analysis research, it is common to use emotion lexicons based on the Emotion Expression Dictionary^43^.

Text analysis was conducted by creating co-occurrence network diagrams. These visualise the connections between words based on their co-occurrence patterns in paragraphs or sentences, indicating the similarity of word occurrence patterns within a text^34^. Automatically detecting words that are associated with each other, categorizing them into groups, and representing the results through color-coding. The size of the circles represents the frequency of word occurrence, and words that co-occur are connected by lines. The thickness of these lines indicates the strength of the co-occurrence, with thicker lines indicating a stronger association. Words grouped in the same color are connected by solid lines, while connections between words outside of the grouping are indicated by wavy lines.

Referring to previous studies^27^, participants were divided into two groups based on their IAcc scores using a cutoff score of 0.85: a high IAcc group (IAcc ≥ 0.85) and a low IAcc group (IAcc < 0.85). Exploratory comparisons were performed between the co-occurrence network diagrams of the two groups.

## Results

### Study I: correlation between IAcc and AF when verbalizing one's own emotions

The scores and the mean and standard deviation for each scale used in the tasks are as shown in Table 1. As a result of correlation analyses, a moderate and significant negative correlation (*r* = − 0.36, *p* = 0.021) was observed between IAcc and the Neuroticism scale of the TIPI-J. Individuals with greater neuroticism tended to have a lower ability to perceive their own interoceptive sensations.

**Table 1:** Sample characteristics.

	All (N = 39)		High IAcc group (*n* = 14)		Low IAcc group (*n* = 25)		Correlation with IAcc	
	*M*	*SD*	*M*	*SD*	*M*	*SD*	*r*	*p*
Preliminary survey								
TIPI-J								
Extraversion	6.87	3.04	6.93	2.84	6.84	3.15	− 0.07	n.s
Agreeableness	10.3	2.07	10.2	1.78	10.4	2.21	0.20	n.s
Conscientiousness	6.67	2.44	7.14	2.85	6.40	2.14	0.17	n.s
Neuroticism	8.97	2.51	8.36	2.77	9.32	2.28	− 0.63	< 0.05
Openness	7.64	2.90	7.93	3.10	7.48	2.77	− 0.36	n.s
TAS-20	46.0	7.52	44.6	5.43	46.8	8.36	0.20	n.s
JART								
Predicted FSIQ	107.2	4.22	107.7	4.23	106.9	4.18		
Predicted VIQ	108.5	4.77	109.1	4.74	108.1	4.74		
Predicted PIQ	104.5	3.18	105.0	3.18	104.3	3.14		
IAcc	0.80	0.11	0.90	0.04	0.74	0.09		

Then, we examined the relationship between IAcc and AF in rating data. According to positive AF, the mean Fleiss' kappa coefficient calculated as positive AF for the rating data was *κ* = 0.24 ± 0.08, with a range of *κ* = − 0.01 to 0.43. There was a weak significant negative correlation between Fleiss' kappa coefficient and IAcc (*r* = − 0.22, *p* = 0.046). Hence, greater IAcc corresponded to a greater positive AF in the rating data. As for negative AF, The mean Fleiss' kappa coefficient calculated as Negative AF for the rating data was *κ* = 0.09 ± 0.08, with a range of *κ* = − 0.01 to 0.36. There was no significant correlation between Fleiss' kappa coefficient and IAcc (*r* = 0.11, *p* = 0.525).

Next, we revealed the relationship between IAcc and AF in free description. According to positive AF, the mean standard deviation calculated as positive AF in the free description data was 0.97±0.23, with a range of 0.40 to 1.40. There was no significant correlation between the standard deviation and IAcc (*r* = 0.03, *p* = 0.844). As for negative AF, the mean standard deviation calculated as Negative AF in the free description data was 1.22 ± 0.32, with a range of 0.31–1.60. There was no significant correlation between the standard deviation and IAcc (*r* = 0.15, *p* = 0.373).

**Figure 2:**
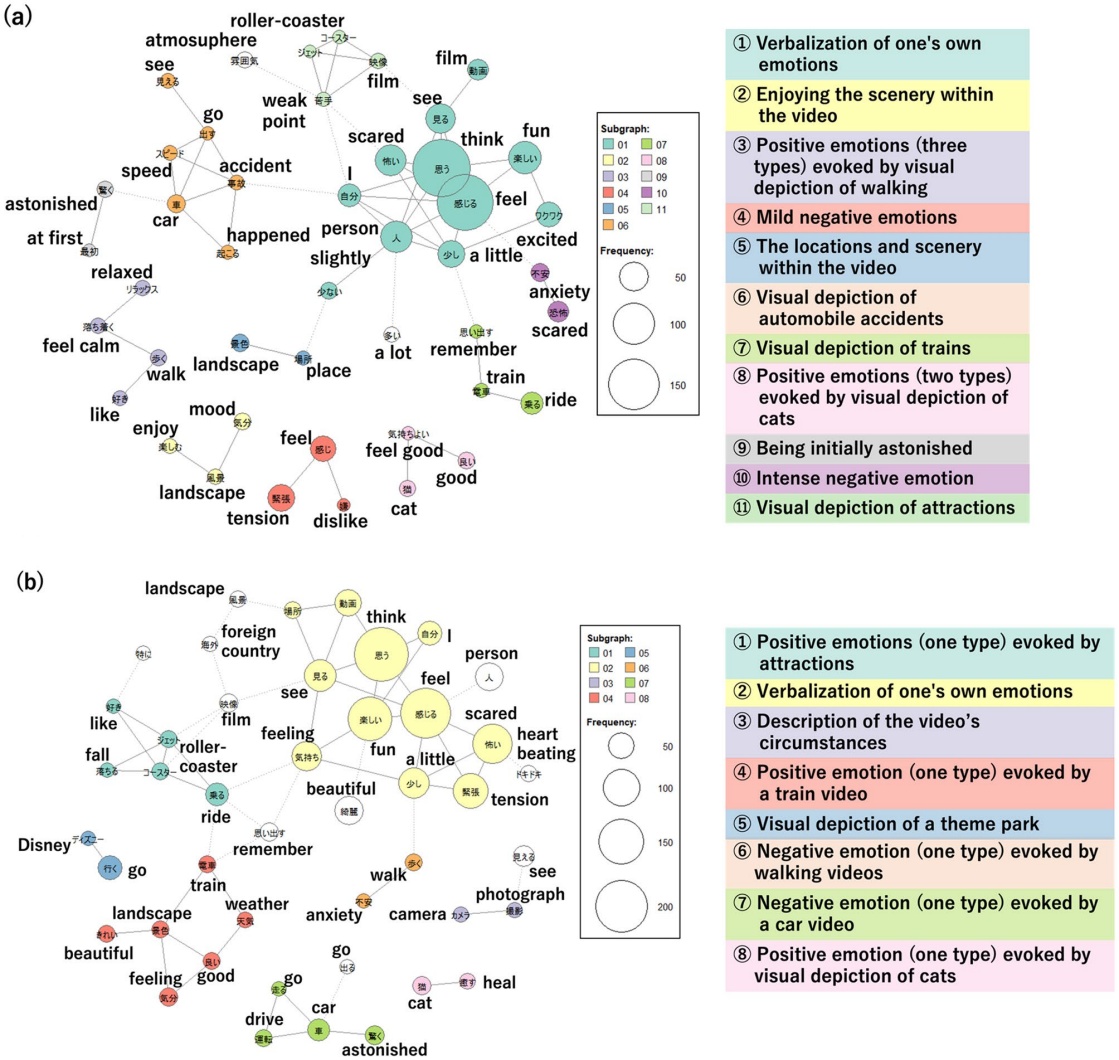
Co-occurrence network diagram in Study I (**a**) high IAcc group, (**b**) low IAcc group. The size of the circles represents the frequency of word occurrence, and words that co-occur are connected by lines. The thickness of these lines indicates the strength of the co-occurrence, with thicker lines indicating a stronger association.

Finally, we conmducted text mining using free description data. The total number of extracted words calculated by KH Coder was divided by the number of days the research participants provided answers, and the average number of extracted words per video was calculated as 28.3 ± 20.6, with a range of 3.6–104.7. There was no significant correlation between the average number of extracted words and IAcc (*r* = 0.07, *p* = 0.666). Therefore, IAcc was not associated with the number of words used in the descriptions. The number of words related to emotions was calculated among the top 150 words in terms of frequency of occurrence. The average number of words related to emotions was 12.1 ± 5.31, with a range of 1 to 23. There was a weak significant positive correlation between the number of words related to emotions and IAcc (*r* = 0.18, *p* = 0.045). That is, greater IAcc was associated with a larger number of words describing emotions. Additionally, there was no significant correlation between predicted verbal IQ (VIQ) and the number of words related to emotions (*r* =0.25, *p* = 0.16). Besides, we explatory examined the co-occurrence network diagrams. The high IAcc group consisted of 14 individuals (5 males, 9 females; mean age 20.7 ± 1.16 years). A co-occurrence network diagram of the high IAcc group is shown in Fig. 2a. The groupings of each category are presented in Table 2(a). In *groups 4*, *9*, and *10*, negative emotions were expressed in a more detailed manner. Additionally, positive emotions were further subdivided based on the type of video, as observed in *Group 3* and *8*. The low IAcc group consisted of 25 individuals (10 males, 15 females; mean age 21.2 ± 1.62 years). A co-occurrence network diagram for the low-IAcc group is shown in Fig. 2b. The groupings of each category are presented in Table 2(b). In contrast to the co-occurrence network diagram of the high-IAcc group, there was only one type of emotion word that co-occurred with each video type, and the emotions were not further subdivided.

**Table 2:** The grouping of the co-occurrence network diagram (a) high IAcc group in study I, (b) low IAcc group in study I, (c) high IAcc group in study II, (d) low IAcc group in study II.

Group number	Label	Contents
(a) High IAcc group in study I		
1	Verbalization of one's own emotions	The association between expressions such as 'think' and 'feel,' and the terms 'enjoyment' and 'excitement'
2	Enjoying the scenery within the video	The association between terms such as 'scenery' and 'enjoyment,' and 'scenery' and 'mood'
3	Positive emotions (three types) evoked by visual depiction of walking	The association between terms such as 'walking' and 'liking,' and 'relaxing' and 'calming down'
4	Mild negative emotions	The association between terms such as 'sensation' and 'nervousness,' and 'sensation' and 'dislike'
5	The locations and scenery within the video	The association between 'location' and 'scenery'
6	Visual depiction of automobile accidents	The association between terms such as 'car' and 'accident,' and 'speed' and 'acceleration'
7	Visual depiction of trains	The association between terms such as 'train' and 'remembering,' and 'train' and 'boarding'
8	Positive emotions (two types) evoked by visual depiction of cats	The association between terms such as 'pleasant' and 'cat,' and 'pleasant' and 'good'
9	Being initially astonished	The association between 'initial' and 'astonishment'
10	Intense negative emotion	The association between 'anxiety' and 'fear'
11	Visual depiction of attractions	The association between terms such as 'jet' and 'coaster,' and 'video' and 'discomfort'
(b) Low IAcc group in study I		
1	Positive emotions (one type) evoked by attractions	The association between terms such as 'roller' and 'roller coaster,' and 'roller' and 'liking'
2	Verbalization of one's own emotions	The association between terms such as 'thinking' and 'feeling,' and 'enjoyable' and 'sensation
3	Description of the video's circumstances	The association between 'camera' and 'photography'
4	Positive emotion (one type) evoked by a train video	The association between terms such as 'train' and 'scenery,' and 'good' and 'mood'
5	Visual depiction of a theme park	The association between 'Disney' and 'going'
6	Negative emotion (one type) evoked by walking videos	The association between 'anxiety' and 'walking'
7	Negative emotion (one type) evoked by a car video	The association between terms such as 'car' and 'driving,' and 'car' and 'surprise'
8	Positive emotion (one type) evoked by visual depiction of cats	The association between 'cat' and 'healing'
(c) High IAcc group in study II		
1	Positive emotions and negative emotions conveyed through the film of interviews	The association between terms such as 'interview' and 'smiles,' and 'nervousness' and 'anxiety'
2	Depiction of the content of the video	The association between terms such as 'self' and 'possess,' and 'perceive' and 'location'
3	Negative emotions evoked by visuals of automobiles	The association between terms such as 'vehicle' and 'driving,' and 'sorrowful' and 'companion'
4	Negative emotions	The association between terms such as 'fear' and 'despair,' and 'frightening' and 'perish'
5	Negative emotions evoked by unsanitary videos	The association between terms such as 'unclean' and 'hand,' and 'mood' and 'unpleasant'
6	The object of verbalization and the verbalization of emotions	The association between terms such as 'males' and 'observe,' and 'enjoyable' and 'perceive'
7	Attributes of the characters	The association between terms such as 'men' and 'women,' and 'individuals' and 'females'
(d) Low IAcc group in study II		
1	The object of verbalization and the verbalization of emotions	The association between terms such as 'females' and 'males,' and 'nervousness' and 'perceive'
2	Depiction of the content of the film and negative emotions	The association between terms such as 'fear' and 'situation,' and 'arrive' and 'contemplate'
3	Positive emotions evoked by visual depiction of cats	The association between terms such as 'cats' and 'time,' and 'enjoyable' and 'happiness'
4	Depiction of the content of the video	The association between terms such as 'videos' and 'appear' and 'now' and 'express'
5	It is delightful to hear one's voice	The association between terms such as 'voice' and 'delighted' and 'voice' and 'listen'
6	Negative emotions evoked by unsanitary videos	The association between terms such as 'unclean' and 'hand' and 'feeling' and 'unpleasant'
7	Negative emotions evoked by visuals of automobiles	The association between terms such as 'driving' and 'car' and 'getting angry' and 'car'
8	Depiction of the content of the film	The association between terms such as 'butter' and 'beer' and 'consume' and 'delicious'
9	Attributes of the characters	The association between terms such as 'companion' and 'lonely' and 'sad' and 'lonely'
10	The negative emotions of the counterpart	The association between terms such as 'person' and 'man'

### Study II: correlation between IAcc and AF when verbalizing inferred emotions of others

The mean and standard deviation of each scale score and task score are the same as in Study I (Table 1). Additionally, the results of the correlation analysis between each scale score and the IAcc were the same as those of Study I.

Then, we examined the relationship between IAcc and AF in rating data. According to positive AF, the average Fleiss' kappa coefficient calculated to represent positive AF in the rating data was 0.32 ± 0.12, with a range of 0.12–0.64. There was a weak and significant negative correlation between Fleiss' kappa coefficient and IAcc (*r* = − 0.14, *p* = 0.048). This suggests that as IAcc increased, positive AF in inferring others' emotions in the rating data also increased. As for negative AF, The average Fleiss' kappa coefficient calculated for negative AF in the rating data was 0.12 ± 0.07, with a range of 0.0002–0.21. No significant correlation was observed between Fleiss’ kappa coefficient and IAcc (*r* = 0.08, *p* = 0.616).

Next, we revealed the relationship between IAcc and AF in free description. According to positive AF, the average standard deviation of positive AF calculated from the free description data was 1.03 ± 0.24, with a range of 0.24–1.60. There was no significant correlation between the standard deviation and IAcc (*r* = − 0.06, *p* = 0.722). As for negative AF, The average standard deviation of negative AF calculated from the free description data was 1.24 ± 0.26, with a range of 0.54–1.60. There was no significant correlation between standard deviation and IAcc (*r* = 0.02, *p* = 0.910).

Finally, as with Study I, we conmducted text mining using free description data. the average number of extracted words per video was calculated as 30.9 ± 24.3, with a range of 4.1–128.2. No significant correlation was observed between the average number of extracted words and IAcc (*r* = 0.14, *p* = 0.401). This suggests that IAcc was not significantly associated with the number of words used in the descriptions. The average number of emotion-related words was 18.2 ± 7.10, with a range of 3– 34. There was no significant correlation between the number of emotion-related words and IAcc (*r* = − 0.07, *p* = 0.674). However, there was a weak significant positive correlation between predicted VIQ (verbal IQ) and the number of emotion-related words (*r* = 0.15, *p* = 0.044). This indicates that individuals with a higher predicted verbal IQ (VIQ) tended to use more emotion-related words when inferring others' emotions.

**Figure 3:**
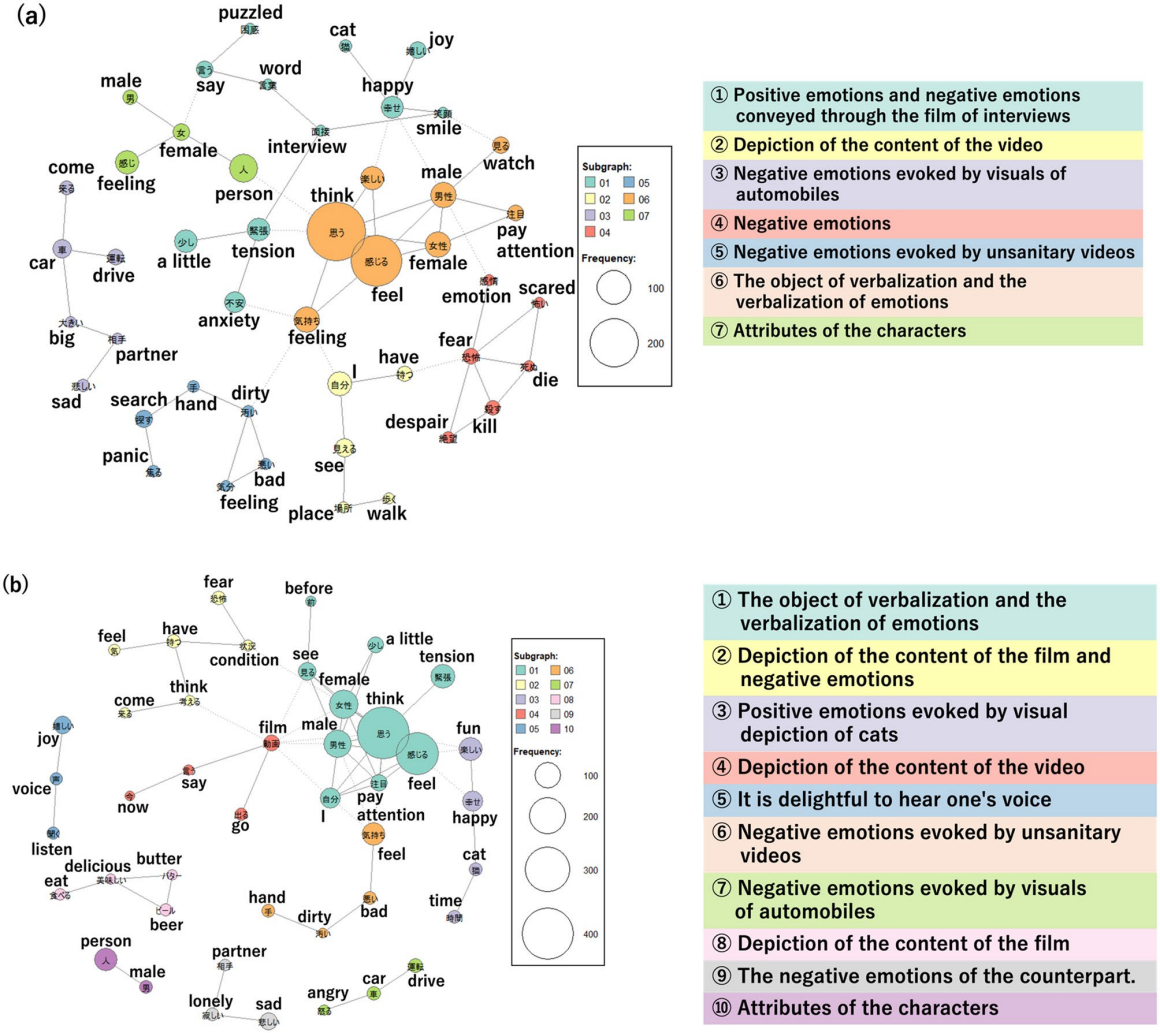
Co-occurrence network diagram in Study I (**a**) high IAcc group, (**b**) low IAcc group. The size of the circles represents the frequency of word occurrence, and words that co-occur are connected by lines. The thickness of these lines indicates the strength of the co-occurrence, with thicker lines indicating a stronger association.

As with Study I, we explatory examined the co-occurrence network diagrams. Similar to Study I, the high IAcc group consisted of 14 participants (5 males, 9 females; mean age 20.7 ± 1.16 years). A co-occurrence network diagram of the high-IAcc group is shown in Fig. 3a. The group categorisation is presented in Table 2(c). There was frequent co-occurrence of specific video descriptions with related emotional words in *Group 1*, *3*, and *5*. However, multiple co-occurrences were observed between subgroups. The IAcc low group consisted of 25 individuals (10 males and 15 females;mean age 21.2±1.62 years). A co-occurrence network diagram of the low-IAcc group is shown in Fig. 3b. The respective categories are listed in Table 2(d). There were subgroups (*Group 8* and *10*) pertaining to the descriptive aspects of video scenarios that did not co-occur with other subgroups. There was a prevalence of situational depictions unrelated to the emotional states of the characters.

## Discussion

### Interoception and neuroticism

In this study, we investigated the relationship between the characteristics of the language used to articulate one's own emotions, others' emotions, and interoception. Although there was no association between personality tendencies and characteristics of words, individuals with a higher inclination toward neurosis exhibited lower levels of IAcc. Neurosis proneness has frequently been associated with maladaptation and has been shown to be positively correlated with emotional and behavioral problems^44^. The identified association with the IAcc suggests that in addition to explaining these issues from a traditional personality perspective, it is plausible to attribute such characteristics to a diminished ability to perceive the interoceptive sensations involved in emotion generation. Students with proneness to neurosis are more susceptible to stress and frequently report physical discomfort^45^. Therefore, it is possible to explain the symptoms and disorders linked to neurosis proneness from an interoceptive perspective.

### The relationship between interoception and verbal expression of self-emotions

In Study I, a significant negative correlation was observed between Fleiss' kappa coefficient for positive emotion adjectives and IAcc. In addition, no significant correlation was found between negative AF and IAcc. Hence, greater IAcc is associated with more positive AF, suggesting that individuals who accurately perceive their interoceptive sensations can assess positive emotions in a more nuanced manner.

This result implies that the ability to accurately perceive the internal bodily sensations that occur when positive emotions are evoked leads to a more differentiated labelling of positive emotion words based on their interoception. This may explain why a greater IAcc is associated with better mental health^46^. Unlike negative emotions, positive emotions have been proposed to broaden thought and behavioural repertoires and enhance individuals' abilities and resources (i.e. broaden-and-build theory)^38^. As a greater IAcc indicates a greater ability to differentiate positive emotions, it leads to various cognitions and behavioural repertoires, and might contribute to better mental health.

The text-mining results revealed a significant positive correlation between the number of emotion-related words and IAcc. That is, greater IAcc was associated with a greater occurrence of emotion-related words during free-text expression. The use of emotional words in spontaneous speech has been linked to activation in the primary and secondary somatosensory cortices, anterior mid-cingulate cortex, and insular cortex^47^. These brain regions also overlap with the neural substrates related to interoception. Therefore, the results of this study are consistent with previous research, demonstrating that the accuracy of interoceptive perception is associated with the use of words to express emotions.

Based on the characteristics of the co-occurrence network diagram, the high IAcc group exhibited finer differentiation of both positive and negative emotions. Conversely, the low IAcc group expressed a relatively fixed set of emotions depending on the type of video content. The correspondence between the specific emotions expressed and the types of videos suggests that individuals in the low IAcc group may verbalise emotions based on those that could be inferred from their environment and context. An association between top-down cognitive processes and interoception has also been reported in previous studies. Tsakiris et al.^48^ investigated this phenomenon using the rubber hand illusion, which involves the illusion of perceiving a rubber hand as one's own hand when both are simultaneously stroked with a brush, highlighting the formation of self-body perception through top-down processes^49,50^. Their study demonstrated that individuals with higher IAcc are less likely to experience the rubber hand illusion, suggesting that higher IAcc suppresses top-down processing, leading to reduced susceptibility to illusions. In the present study, it is conceivable that individuals with low IAcc verbally expressed emotions through top-down processes, whereas those with high IAcc employed a bottom-up process that integrated their interoceptive awareness when verbalising emotions.

### Relationship between interoception and verbalization of others' emotions

In Study II, similar to the results of Study I, no significant correlations were found between positive AF and negative AF in the free description data and IAcc. However, analysis of the rating data revealed that greater IAcc corresponded to more positive AF. This may be related to the tendency to prioritise the processing of happy facial expressions over other emotions. Calvo et al.^51^ demonstrated that happy faces are detected more quickly than are faces expressing fear, anger, sadness, surprise, or disgust. Moreover, individuals with greater IAcc are quicker at recognising emotions from others' facial expressions^5^. Therefore, individuals with greater IAcc may recognise happy facial expressions more rapidly, leading to greater verbalisation of positive emotions with different levels of arousal. However, the extent to which the facial expressions of the characters in videos play a crucial role in observers’ verbalising of the perceived emotions and individual differences in the importance of these factors remain unclear. Further investigation considering these aspects would be valuable.

Furthermore, text mining revealed a significant positive correlation between the number of emotion-related words and predicted verbal IQ (VIQ), contrary to the findings of Study I. That is, individuals with better linguistic abilities tended to employ a larger number of emotional words when verbalising others’ emotions. When inferring the emotions of others, careful attention must be paid to various environmental factors such as facial expressions, tone of voice, and contextual cues. Consequently, the influence of a bottom-up process, wherein emotions arise through the perception of one's internal receptivity, can be diminished when inferring the emotions of others. Hence, the influence of the top-down process, which depends on an individual's linguistic ability, such as the expectation that this emotion word would be appropriate in this situation, would be greater.

As evidenced by these findings, the co-occurrence network diagrams suggested a tendency within the low-IAcc group to specialise in describing video content rather than portraying the emotions of individuals appearing in those videos. Additionally, both the high and low IAcc groups exhibited subgrouping of content descriptions and emotion words within the videos. In the low IAcc group, attention appeared to be directed towards external aspects such as describing the content of the videos. From these results, it can be concluded that individuals possess knowledge of emotions such that it is common to feel certain emotions in this context, regardless of the degree of IAcc. Knowledge of these emotions is influenced by previous opportunities for interpersonal interaction. Therefore, the ability to infer and verbalise the feelings of others appears related to top-down processes such as linguistic competence and an individual's previous learning experiences in interpersonal interactions.

## Limitations

In this study, we revealed that greater IAcc would be related to more positive AF. However, this study have a few limitations. First of all, only results with a low level of significance were observed in this study. We did not correct for multiple comparisons and it is also an limitation regarding the analysis. Given the small effect size, although this study reveals a statistically significant association, it is possible that there may be no inherent correlation. In the future, it is considered necessary to increase the number of data and conduct further analysis.

Secondly, with regard to the relationship between IAcc and positive AF observed in this study, it should be noted that the mechanism is not yet fully understood. In this study, we were able to demonstrate the relationship between IAcc and positive AF at the behavioral level, which had not been examined previously. However, in the future, it will be necessary to gain insights from a neuroscientific perspective to explain the mechanism behind why these are related.

Besides, although analysis of the free description data revealed no significant correlation between positive and negative AF or IAcc, this result may be attributed to the influence of the text mining methodology. For the free description data, AF scores were calculated for each individual sentence, yielding separate positive and negative scores. That is, when multiple emotion-related words appeared in a sentence, they were aggregated into single values that potentially offset each other. Subsequent text-mining findings suggested that greater IAcc was associated with the use of emotion-related words. Therefore, when aggregated into a single value, the relationship with IAcc may become less discernible, possibly contributing to the lack of a significant correlation. In the future research, it could be possible to calculate the variability of scores within a single sentence, rather than computing positive and negative scores for individual sentences and examining the standard deviation of the entire dataset.

In addition, the participants were asked to express their own feelings about the video stimuli in written form. While this method offers advantages in stimulus control and ease of data collection, it diverges from everyday life, in which face-to-face communication is more common than communication through videos. Furthermore, the relationship between the verbalisation of emotions in speech-like formats, such as conversation, and interoceptive awareness was not elucidated by this study. Future research should investigate the relationship between verbalisation of emotions and interoceptive awareness in settings that resemble daily life situations, such as conversational contexts.

Finally, in Study II, the participants inferred the emotions of unfamiliar individuals depicted in videos. The inferral could vary according to the individual observed, such as close family members, friends, or individuals with whom participants have a negative impression. Future research should consider the complexities of interpersonal interactions in such contexts.

### Clinical implications

Lieberman et al. (2007)^52^ considered the verbal expression of emotions as ‘affect labelling’ and treated labelling as an unintentional emotional regulation strategy. Such strategies are associated with mental well-being; for instance, labelling positive emotions can effectively amplify them^53^. The current study elucidated the relationship between interoception and language. Consequently, it is anticipated that enhancing one's sensitivity to interoception will enable a more nuanced verbalisation of one’s positive emotions, which, in turn, could potentially contribute to one's mental well-being. Moreover, these findings indicate a higher-order function linking language, a social construct, to bodily responses and perceptual abilities, thereby illustrating the relationship between human embodiment and sociability.

Study II indicated that individuals with high IAcc demonstrated a greater ability to infer and verbalise positive emotions from others, resulting in more detailed expressions and increased usage of emotional vocabulary. This finding represents a valuable insight, particularly for individuals functioning as therapists who engage in psychotherapeutic interviews utilising language as a tool.

During psychotherapeutic interviews, the focus is often on exploring the clients’ emotions through verbal expressions. However, owing to various factors such as symptoms, it can be challenging for individuals to articulate their emotions in words. In such cases, developing psychotherapy interviews may be particularly difficult. For instance, clients experiencing depressive states commonly exhibit pessimistic cognition, which makes it challenging for them to identify positive emotions in their own lives. Alexithymia, characterised by difficulties in recognising emotions, is a clinically observed phenomenon that extends across diagnostic boundaries. Individuals with dementia often display limited emotional expression and numbness.

However, based on the findings of this study, it can be inferred that therapists with high IAcc are capable of expressing positive emotions related to the client, as conveyed through their content and facial expressions, using diverse vocabulary while also delineating the magnitude of these emotions. Receiving such feedback from therapists is likely to facilitate a client's deepening of self-understanding. While consistent findings are yet to be established, it has been demonstrated that IAcc can be enhanced through interventions^27,54^. Thus, it can be argued that improving the accuracy of one's perception of interoceptive sensations not only enhances one's own mental well-being but also has the potential to enhance the mental well-being of others. Furthermore, these findings can serve as supportive indicators of the significance of self-understanding among therapists in clinical settings.

## Data Availability

The datasets generated and/or analyzed during the current study are available from the corresponding author on reasonable request.
